# Supplementary material for: Parallel triplet formation pathways in a singlet fission material
Source: Nat Commun. 2022 Sep 6;13:5244. doi: 10.1038/s41467-022-32844-6 (PMC9448805; doi:10.1038/s41467-022-32844-6)
Supplement: Supplementary file 1 — Supplementary Information [file 41467_2022_32844_MOESM1_ESM.pdf]

Supplementary Information:

Parallel triplet formation pathways in a singlet fission material

Nilabja Maity[1,†], Woojae Kim[2,†,‡], Naitik A. Panjwani[3], Arup Kundu[4], Kanad Majumder[1], Pranav Kasetty[1], Divji Mishra[1], Robert Bittl*[3], Jayashree Nagesh*[1], Jyotishman Dasgupta*[4], Andrew J. Musser*[2], and Satish Patil*[1]

[1]Solid State and Structural Chemistry Unit, Indian Institute of Science, Bangalore 560012, India

[2]Department of Chemistry and Chemical Biology, Cornell University, Ithaca, New York 14853, USA

[3]Berlin Joint EPR Lab, Fachbereich Physik, Freie Universität Berlin, 14195 Berlin, Germany

[4]Department of Chemical Sciences, Tata Institute of Fundamental Research, Mumbai 400005, India

[†]These authors contributed equally: Nilabja Maity, Woojae Kim.

[‡]Present Address: Department of Chemistry, Yonsei University, Seoul 03722, Republic of Korea

Correspondence to: robert.bittl@fu-berlin.de, jayashreen@iisc.ac.in, dasgupta@tifr.res.in, ajm557@cornell.edu, spatil@iisc.ac.in

Supplementary Note 1. Synthetic details

All solvents were dried by standard methods. Chemicals were purchased from Aldrich, Acros Organics, S.D. fine chemicals, and Spectrochem and used without further purification. HR-TDPP-TEG was synthesized via Sonogashira coupling using palladium $Pd(PPh_3)_4$.

The ^1H spectra were taken in d_8-THF in Bruker Advance NMR spectrometer at 400 MHz frequency. The chemical shifts were reported as δ values (ppm) relative to TMS. Also, ^{13}C were recorded on the same instrument at 100 MHz frequency.

MALDI-MS were recorded on a Bruker daltonics Autoflex Speed system using α-Cyano-4-hydroxy-cinnamic acid (CCA) as a matrix.

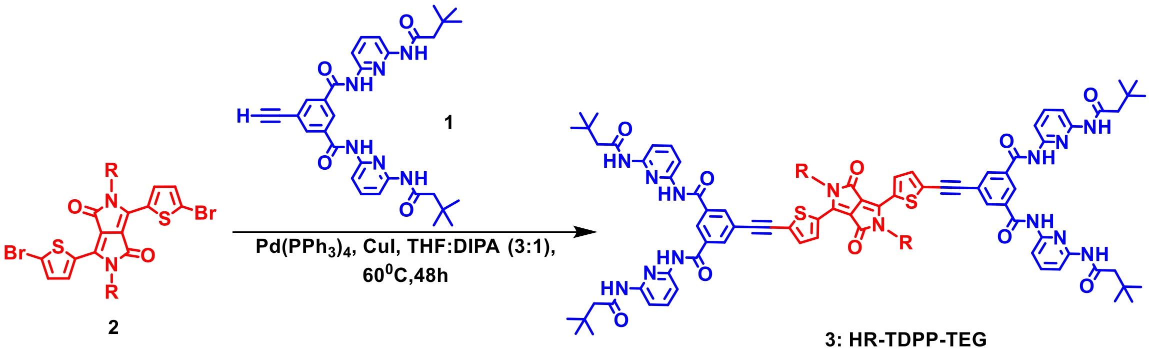

Supplementary Fig. 1 Synthetic Scheme of HR-TDPP-TEG.

Synthesis of HR-TDPP-TEG (3): In a 250 mL double necked round bottom flask with a magnetic stirring bar, equipped with Ar bladder, N,N'-Bis[6-(3,3-dimethylbutyrylamino)pyridine-2-yl]-5-ethynyl-isophthalamide (1), (500mg, 0.88 mmol, 2.5 eqv), corresponding di-bromo derivatives of TDPP-TEG-Br (2) (1 eqv), $Pd(PPh_3)_4$ (0.1 eqv, 10%) and CuI (0.2 eqv, 20%) were taken in dry THF and diisopropylamine mixture (3:1; v/v) of 30 ml, stirred and heated at 60°C for 48h in Ar atmosphere. The resulting mixture was washed thoroughly three times with a saturated solution of Na_2EDTA. The organic phase was extracted with CH_2Cl_2 (3 × 50 mL), washed with water and dried over anhydrous Na_2SO_4. crude mixture obtained was purified with silica gel chromatography eluting with chloroform: methanol (2-3% methanol). After that, the product was further ultra-purified by preparative TLC using a similar solvent system to afford a deep blue isolated. Yield: 35%

Spectroscopic characterization of HR-TDPP-TEG (3):

^{1}H NMR (400 MHz, d_8-THF, rt): δ 9.66 (br s, 4H), 9.03 (br s, 4H), 8.86 (br s, 2H), 8.43 (s, 2H), 8.19 (br s, 4H), 8.01-7.79 (t, 8H), 7.71-7.67 (t, 4H), 7.42 (br s, 2H), 4.31 (s, 4H), 3.81 (br s, 4H), 3.53-3.51 (m, 4H), 3.49-3.46 (m, 4H), 3.39-3.37 (m, 4H), 3.22 (br s, 4H), 2.29 (br s, 8H), 1.10 (br s, 42H). d_8-THF solvent peak at 3.58 and 1.72 ppm, water 2.55 ppm; ^{13}C NMR (100 MHz, d_8-THF, rt): δ 168.09, 161.97, 158.82, 148.99, 148.41, 137.80, 131.27, 121.15, 107.69, 107.47, 70.03, 68.85, 68.61, 68.54, 67.12, 56.10, 48, 28.92, 27.29, 24.92; MALDI-TOF (m/z): $[M]^+$ calcd. for $C_{92}H_{104}N_{14}O_{16}S_2$, 1724.719; found, 1724.848; analysis (calcd., found for $C_{92}H_{104}N_{14}O_{16}S_2$): C (64.02, 63.86), H (6.07, 6.08), N (11.36, 11.25), S (3.71, 3.75).

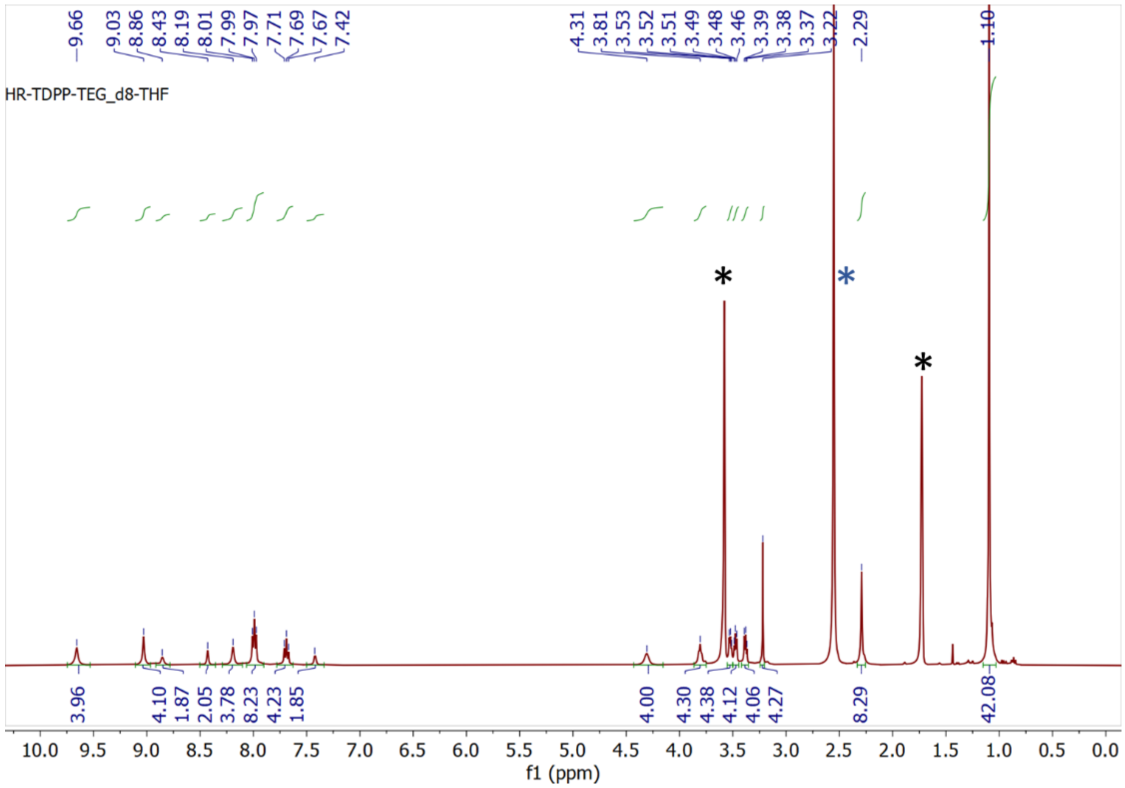

Supplementary Fig. 2 ^{1}H-NMR Spectrum of HR-TDPP-TEG in d_8-THF. The asterisks indicate proton signals from d_8-THF (black) and water (blue).

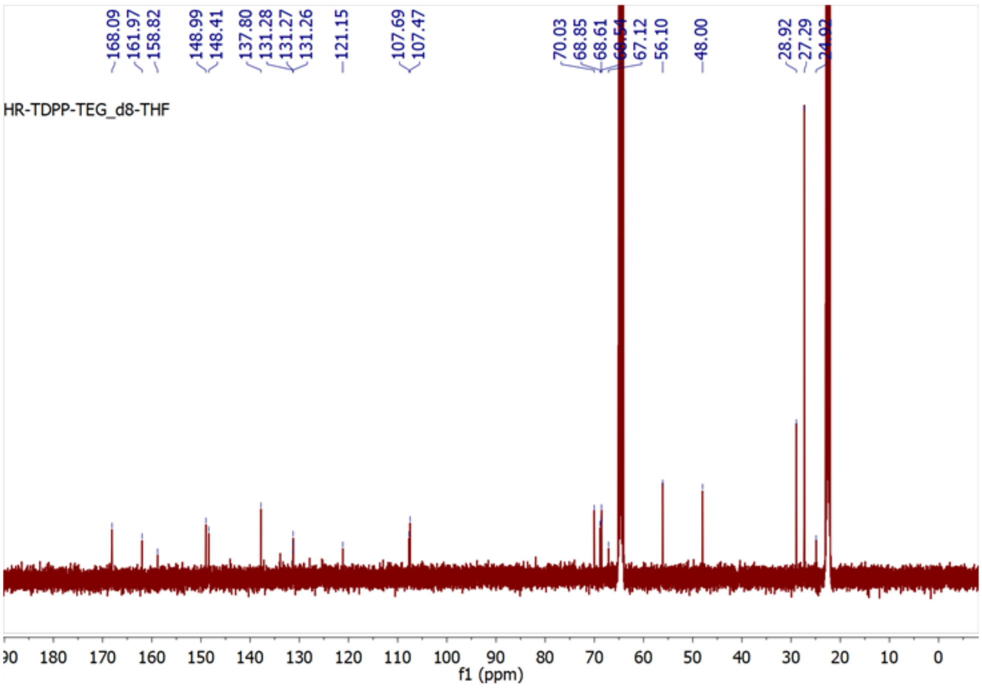

Supplementary Fig. 3 ^{13}C NMR Spectrum of HR-TDPP-TEG in d_8-THF.

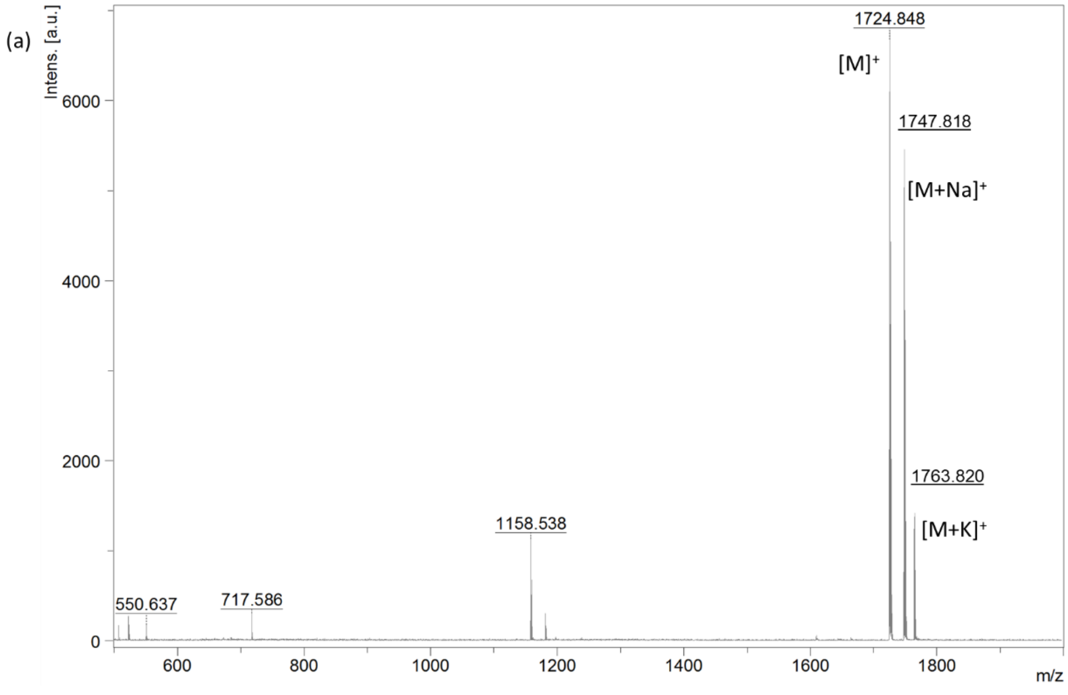

Supplementary Fig. 4 a MALDI-MS spectrum of HR-TDPP-TEG.

b

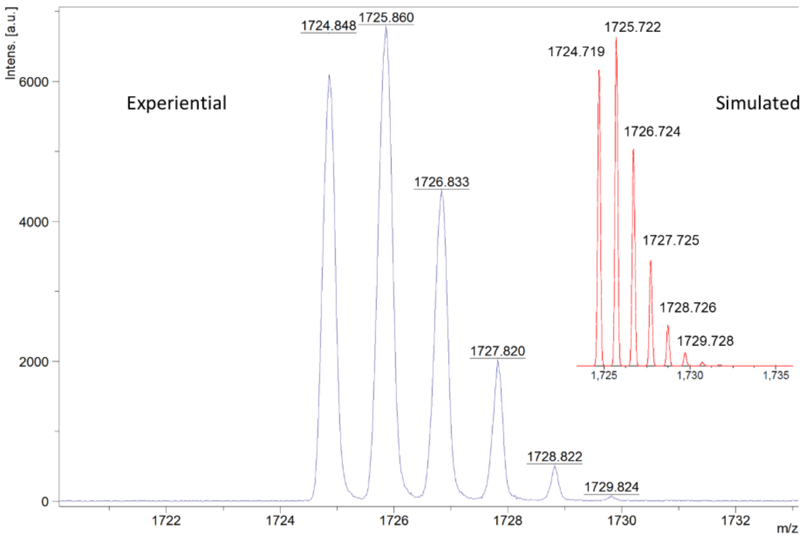
Intens. [a.u.]
Experiential
Simulated
1724.848
1725.860
1726.833
1727.820
1728.822
1729.824
1724.719
1725.722
1726.724
1727.725
1728.726
1729.728
m/z

c

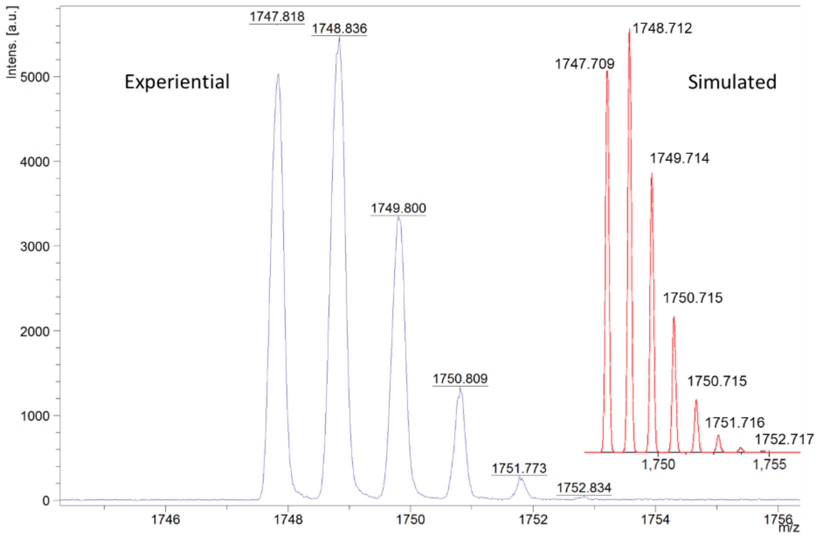
Intens. [a.u.]
Experiential
Simulated
1747.818
1748.836
1749.800
1750.809
1751.773
1752.834
1747.709
1748.712
1749.714
1750.715
1750.715
1751.716
1752.717
m/z

d

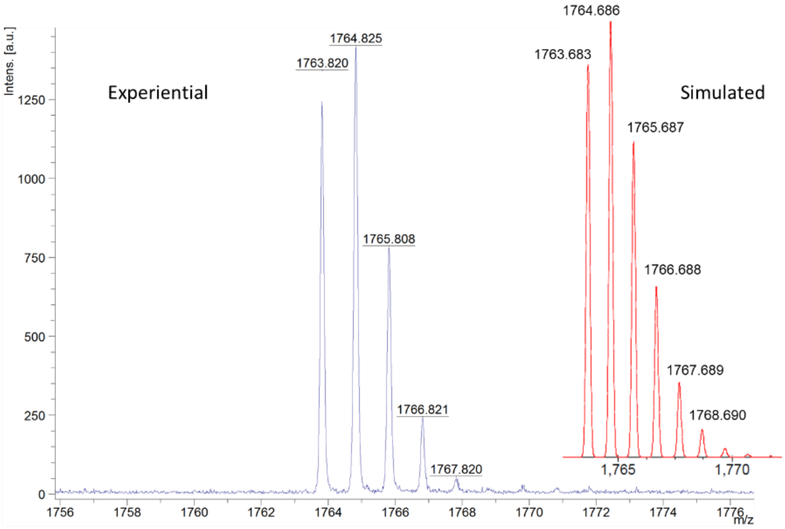
Intens. [a.u.]
Experiential
Simulated
1763.820
1764.825
1765.808
1766.821
1767.820
1763.683
1764.686
1765.687
1766.688
1767.689
1768.690
m/z

Supplementary Fig. 4 (continued) the isotope distributions of **b** $[M]^+$, **c** $[M+Na]^+$ and **d** $[M+K]^+$ peaks for HR-TDPP-TEG. Left panel shows the zoom image of MALDI-TOF experiment and right panel is for simulated isotope distribution of corresponding peak.

Supplementary Note 2. Theoretical Calculation

In this section we discuss the model Hamiltonian used to simulate the aggregate spectra observed upon casting the HR-TDPP-TEG molecules on a thin film and also the ab initio methods used to generate the monomer spectra. To generate the monomer spectra, we first obtained various nuclear configurations corresponding to different electronic energy minimum geometries, namely S_0 and S_1 (the lowest bright state) at TD-B3LYP and TD-CAM-B3LYP level of theory and 6-31G(d) basis set using the Gaussian program[1-3]. The basis set size is kept nominal due to the large number of atoms (228). The presence of only real frequencies confirmed that these were indeed minima on the respective potential energy surfaces (PESs). The B3LYP functional is chosen for further calculations based on its more accurate estimate of the vertical energy from the experimental steady-state spectra (Supplementary Fig. 5 and Supplementary Table 1). Here, the vertical energy from experiment is estimated using the lineshape function.[4] The S_0 and S_1 minima along with their respective set of normal modes and frequencies are used to calculate the vibronically resolved spectra within the Franck-Condon[5] and harmonic approximations[6], taking into account the shift of the minima as well as the Duschinsky effect[7] (rotation of the normal modes). The optimized geometries are provided in a separate zip file.

Supplementary Table 1. Comparison of vertical energies estimated from experiments and calculations.

Functional	E_{vert}(expt)-E_{vert}(TD) (eV)	E_{vert}(expt)-E_{vert}(TDA) (eV)
B3LYP	0.059	-0.118
CAM-B3LYP	-0.285	-0.460

To simulate the aggregate spectrum, we adopt the site-based one-dimensional Frenkel-Holstein Hamiltonian where the nuclear PES for the molecular vibrations (or normal modes) in monomer S_0 and S_1 are shifted harmonic oscillator wells of identical curvature. A vector subspace containing a single electronic excitation within an aggregate consisting of N HR-TDPP-TEG molecules is considered. Within this vector subspace, the Frenkel-Holstein Hamiltonian is given by[8]

$$H = \hbar\omega_o \sum_n b_n^\dagger b_n$$
$$+ \hbar\omega_0\lambda \sum_n (b_n^\dagger + b_n)|n\rangle\langle n| + \sum_{m,n} (J_{mn} + \delta_{mn}D)|m\rangle\langle n| + \mathrm{E}_{00} + \hbar\omega_0\lambda^2 \sum_n |n\rangle\langle n|$$
$$+ \sum_n \Delta_n |n\rangle\langle n|$$

where the ladder operators $(b_n, b_n^\dagger)$ are used for denoting vibrational motion with ω_0 angular frequency, the projection operators ($|n\rangle\langle n|$) refer to the electronic degree of freedom, denoting a single electronic excitation on the nth site with rest of the sites being in their electronic ground states and λ^2 is the Huang-Rhys factor quantifying the shift between S_0 and S_1 minima. The first term represents the total vibrational energy, the second term is the linear coupling between the electronic and vibrational degrees of freedom, the third term is the purely electronic coupling between any two chromophores n and m such that the onsite term is an empirical parameter denoting monomer-to-aggregate shift (D) and the nonequivalent indices will turn on the excitonic coupling J, the E_{00} is the difference in energy between S_1 and S_0 including the zero point vibrational energies, the penultimate term is the total nuclear reorganization energy in S_1 arising due to the shift between the S_1 and S_0 minima and the last term denotes a disorder parameter in the onsite energies (due to static structural inhomogeneity) treated as a random Gaussian variable with a distribution width of σ_d.[6] D is an empirical parameter that is traditionally written as a gas-to-crystal shift, however, in most analysis, the monomer is suspended in a solution and the aggregates are formed on a thin film; hence the monomer-to-aggregate shift is really a solution-to-thin film shift.[8] In the current work we denote D as simply the shift included in the difference between the experimental and the simulated monomer spectra. The effect of D and systematic error from the ab initio calculations is taken together and simply denoted as D for the sake of simplicity. The basis set to solve the above Hamiltonian consists of one- and two-particle states[10]; we neglect higher-particle states in the current work based on our current estimate of S_0-S_1 gap and nuclear relaxation energy in S_1.[11,12] The α^{th}eigenstate is expanded as

$$|\psi_\alpha\rangle = \sum_{n,\tilde{v}} c_{n,\tilde{v}}^{\alpha} |n, \tilde{v}\rangle + \sum_{n,\tilde{v},n',v'} c_{n,\tilde{v},n',v'}^{\alpha} |n, \tilde{v}, n', v'\rangle$$

where the one-particle basis state $|n, \tilde{v}\rangle$ represents electronic excitation on n^{th} chromophore with $\tilde{v}$ vibrational quanta in the S_1 potential well and the two-particle basis state $|n, \tilde{v}, n', v'\rangle$ represents, in addition to the nth chromophore being electronically and vibrationally excited, additional vibrational excitation of v' vibrational quanta on the chromophore n' in its S_0 potential well. The remaining N-2 chromophores are in their vibrationless ground states. The coefficients are obtained by numerically diagonalizing the Hamiltonian. The eigensolutions are used to calculate the room temperature absorption and emission line shape spectra including

the effects of disorder by following the Knapp's model[9], where the set of energy shifts for each site (Δ_n) is chosen from a spatially uncorrelated distribution

$$P(\Delta_1, \Delta_2, \ldots\ldots \Delta_N) = \Pi_{i=1}^{N} p(\Delta_i),$$

where $p(\Delta_i) = \left(\sqrt{(2\pi)\sigma_d^2}\right)^{-1} \exp\left(-\frac{\Delta_i^2}{2\sigma_d^2}\right)$ is the Gaussian distribution for each site. The absorption and emission spectral line shapes are obtained as an average over temperature and disorder as[9]

$$A(\omega) = \left\langle \sum_{\nu=0,1,\ldots} \frac{e^{-\frac{\nu\hbar\omega_{vib}}{k_B T}}}{Z} \sum_{\alpha=0,1,2\ldots} I^{\alpha-\nu} \Gamma(\hbar(\omega - (\omega_\alpha - \nu\omega_{vib}))) \right\rangle_C ,$$

$$I(\omega) = \left\langle \sum_{\alpha=0,1,\ldots} \frac{e^{-\frac{(E_\alpha - E_0)}{k_B T}}}{Z} \sum_{\nu_t=0,1,2\ldots} I^{\alpha-\nu_t} \Gamma(\hbar(\omega - (\omega_\alpha - \nu_t\omega_{vib}))) \right\rangle_C \text{ and}$$

$$I^{\alpha-\nu_t} = \frac{1}{\mu^2} \sum_{\{\nu_t\}} |\langle \psi_\alpha | \hat{\mu} | S(\{\nu_t\}) \rangle|^2$$

where$I^{\alpha-\nu_t}$ is called the linestrength for a given vibronic transition, μ is the magnitude of the transition dipole moment from S_0 to S_1 for the monomer (assumed to be a constant based on the Franck-Condon approximation), $S(\{\nu_t\})$ stands for a set of states with same number of total vibrational quanta ν_t in the final state; the final state is one where all the chromophores are in their electronic ground states, with $\{\nu_t\}$ denoting the set of all monomer vibrational states whose vibrational quanta add up to a total of ν_t, ν stands for number of ground state vibrational quanta in a monomer, ψ_α and $E_\alpha (\equiv \hbar\omega_\alpha)$ represent the αth eigenstate and the corresponding eigenvalue respectively, and $\Gamma(\omega)$ is a Gaussian broadening function $\exp\left(-\frac{\omega^2}{\sigma_g^2}\right)$. The average over temperature is performed by weighting the line strengths with the appropriate Boltzmann factors and the average over configurations is denoted by $\langle \ldots\ldots \rangle_C$.

The experimental spectra are tabulated on a wavelength scale. The simulated spectra are line shapes as a function of energy (in eV) or in wavenumbers. To compare the two, we convert the experimental curves to line shape functions as a function of wavenumbers as follows[12]

$$L_{abs}(\nu) = \frac{N_{norm}^{abs}}{\nu} A_{abs}(\lambda)$$

for absorption, where $A_{abs}(\lambda)$ represents the measured absorbance, ν is the frequency (c/λ) and

$$L_{emi}(\nu) = \frac{N_{norm}^{emi}}{\nu^5} I_{emi}(\lambda)$$

for emission, where $I_{emi}(\lambda)$ is the number of photons emitted per second as a function of wavelength λ; c is the speed of light.

The Frenkel-Holstein vibronic excitonic model works primarily based on the choice of four parameters: J_{mn}, ω_{vib}, λ^2 and N. Based on this framework, the effect of temperature and disorder is introduced to further elucidate their effects on the vibronic signatures of the aggregates. In the current work, since we were unable to obtain a crystal structure of HR-TDPP-TEG using neither chloroform nor THF, we have estimated the Hamiltonian parameters as follows: Yet to be published (submitted for peer review) crystal structure of a similar molecule with Hamilton receptors reveals that molecule crystallizes in a trimeric structure with trimers well-separated from each other, and since the backbone is essentially same as HR-TDPP-TEG, we choose N to be three. An estimate of ω_{vib} is made from the spacing between the peaks observed in the solution spectra believed to consist of mostly monomers, assuming that the spectra are dominated by the vinyl/ring C-C=C stretching mode. The Huang Rhys factor λ^2 is tuned to fit the experimental absorption spectrum of chloroform thin film and the same value is used for the rest. Lastly, the estimate of the intersite coupling J_{mn} is obtained by considering the ratio of the areas under A_1 and A_2 of the thin film spectrum (where aggregation is expected) assuming the perturbative limit ($J_{mn} \leq 0.25\, \lambda^2 \omega_{vib}$).[14] The obtained parameters are listed in Supplementary Table 2, and any modifications to the parameters that are carried out to fit the experimental spectrum better are explained in the Results section. The experiments are carried out at room temperature (T=300K). The monomer ab initio calculations give us an estimate of $\omega_{00} = 12935\, cm^{-1}$ for chlorobenzene. The simulated (monomer and aggregate) spectra are shifted so that the 00 peak aligns with the 00 experimental peak. Any energy shift contribution from the solution to the thin film is assumed to be contained in the aforementioned shift of the whole spectrum to align the 00 peaks. The role and magnitude of disorder is discussed for specific cases in the results section.

Supplementary Table *2*. Parameters used in the simulation of thin film spectra in Fig. 1 *in the main text*. The numbers in the parenthesis for J_{mn} column are obtained from ratio of first two absorption peak areas from thin film (Supplementary Fig.7).

Figure	N	T (K)	λ^2	ω_{vib} (eV)	J_{mn}(meV)	σ_d (meV)	σ_g(cm^{-1})
1b (Film1, absorption)	3	300	0.90	0.19	-51 (-80)	0.0	740
1b (Film1, emission)	3	300	0.90	0.14	-0.03	0.0	740
1b (Film 2, absorption)	3	300	0.90	0.19	80	38	996

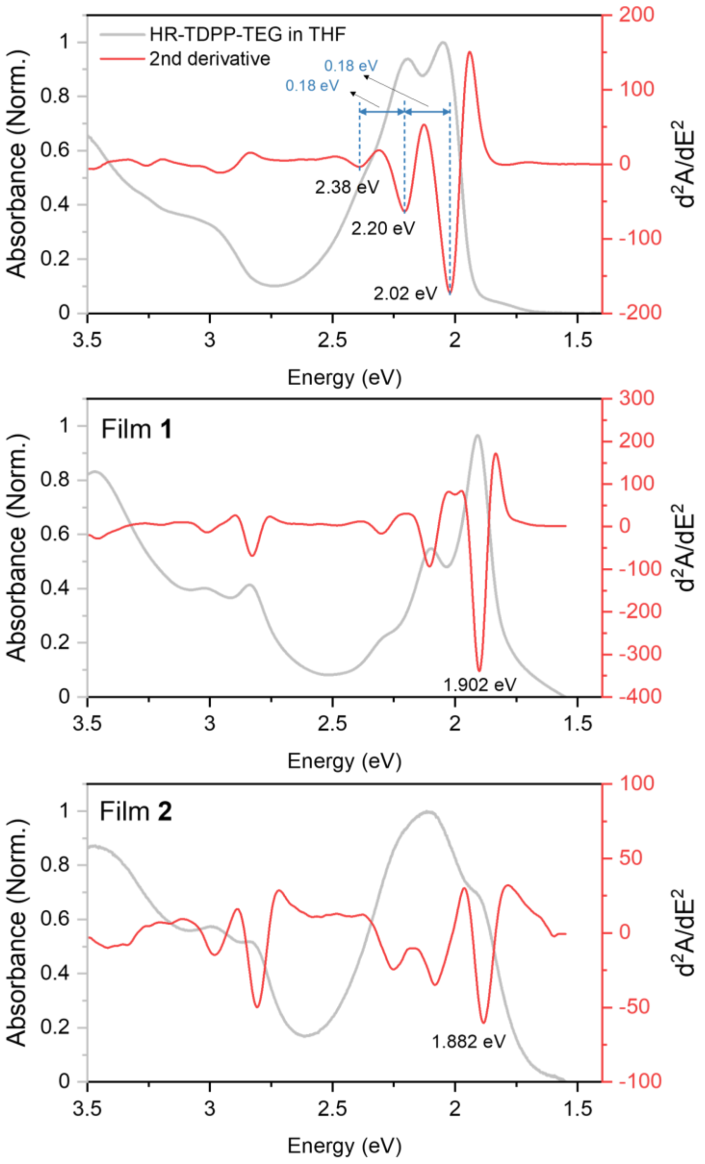

Supplementary Fig. 5 Second derivative analysis for steady-state absorption spectra of monomeric HR-TDPP-TEG in THF (top), film **1** (middle), and film **2** (bottom).

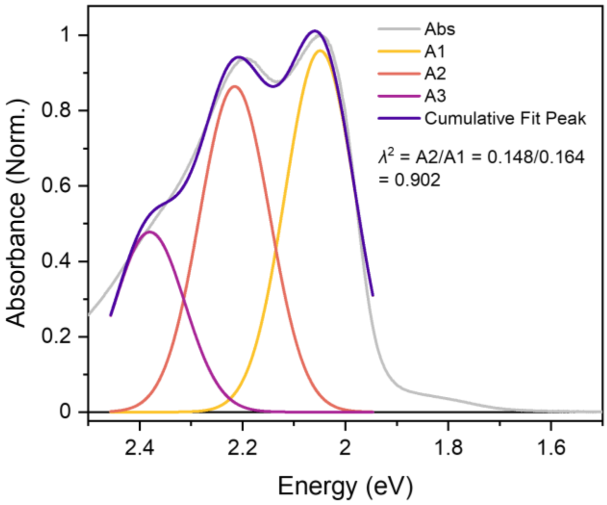

Supplementary Fig. 6 Gaussian fits for the steady-state absorption spectrum of monomeric HR-TDPP-TEG in THF. The estimated Huang-Rhys factor is indicated right next to the fits.

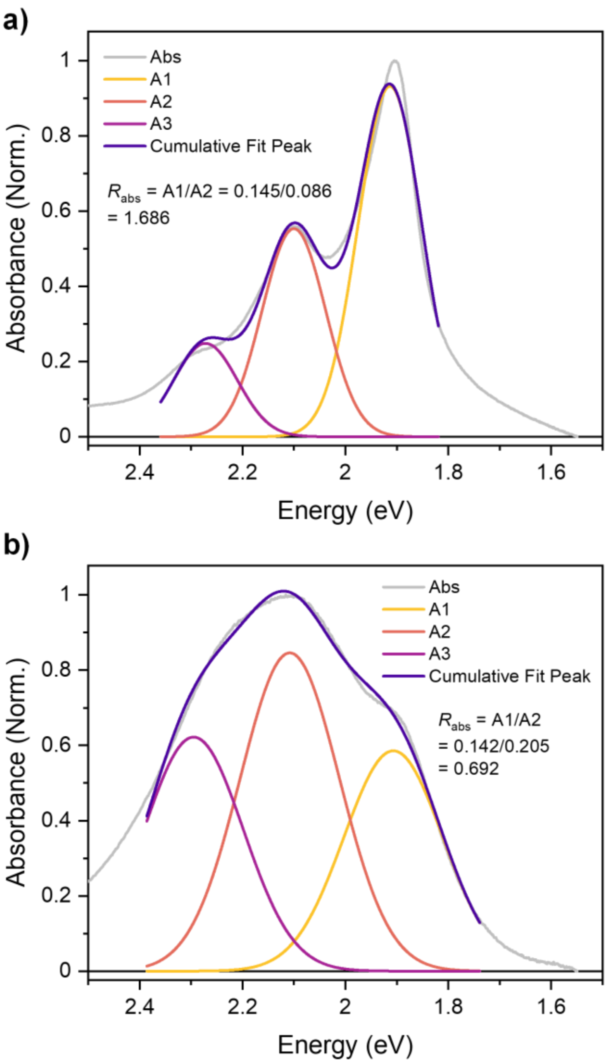

Supplementary Fig. 7 Gaussian fits for the steady-state absorption spectra of **a** J-like film 1 and **b** H-like film 2. The estimated absorption peak ratio values (R_{abs}) are indicated right next to the fits.

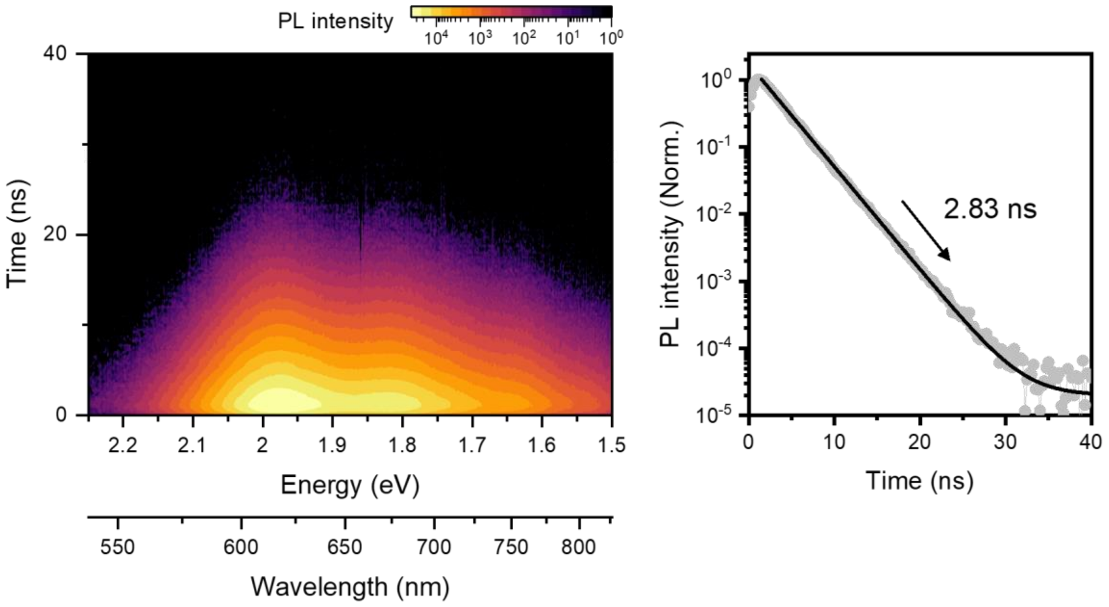

Supplementary Fig. 8 Time-resolved PL spectra (left) and kinetics (right) of monomeric HR-TDPP-TEG in THF.

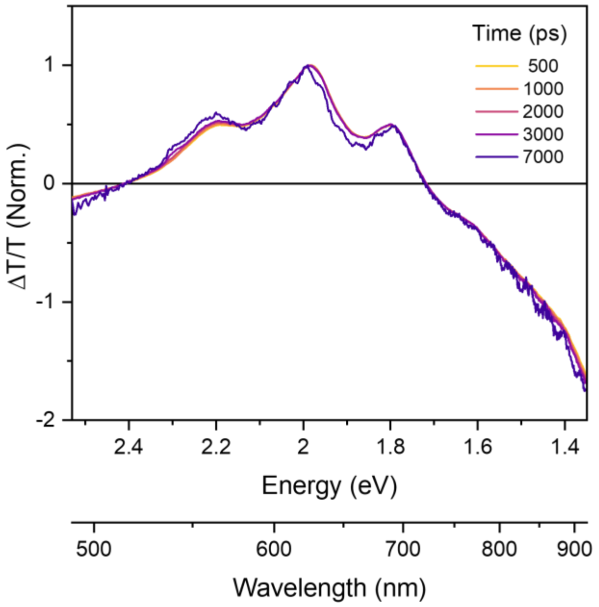

Supplementary Fig. 9 Normalized TA spectra of monomeric HR-TDPP-TEG in THF from 500 to 7000 ps after the structural relaxation process in the S_1 state is done. The perfect overlaps of all spectra indicate no contribution of triplet species.

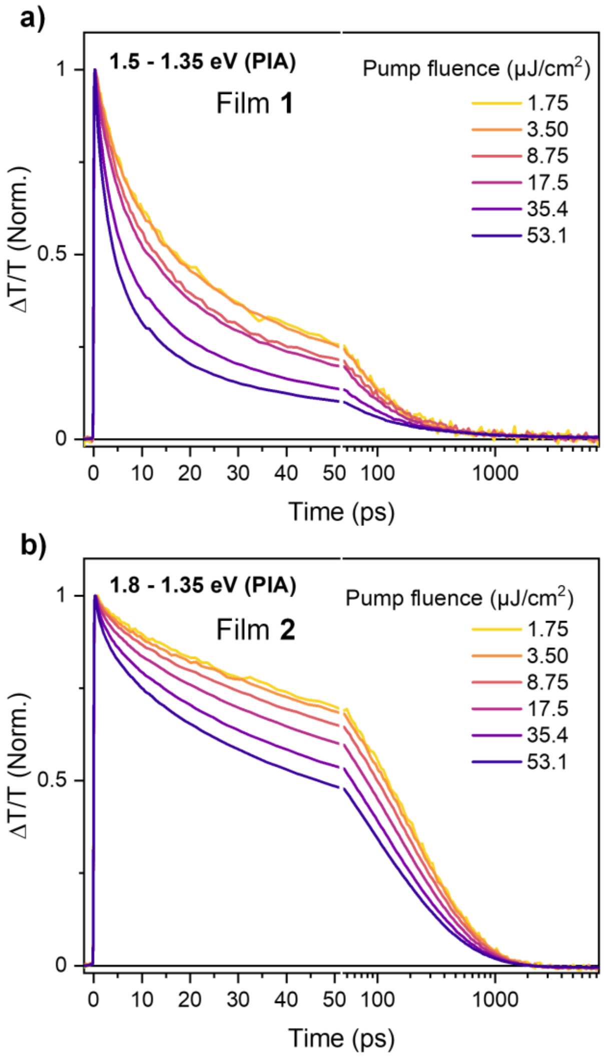

Supplementary Fig. 10 Pump-fluence-dependence of near-infrared PIA kinetics of **a** film 1 and **b** film 2. For both films, there is a negligible contribution of annihilation of singlet excitons when the pump fluence less than 3.50 μJ/cm^2 is used.

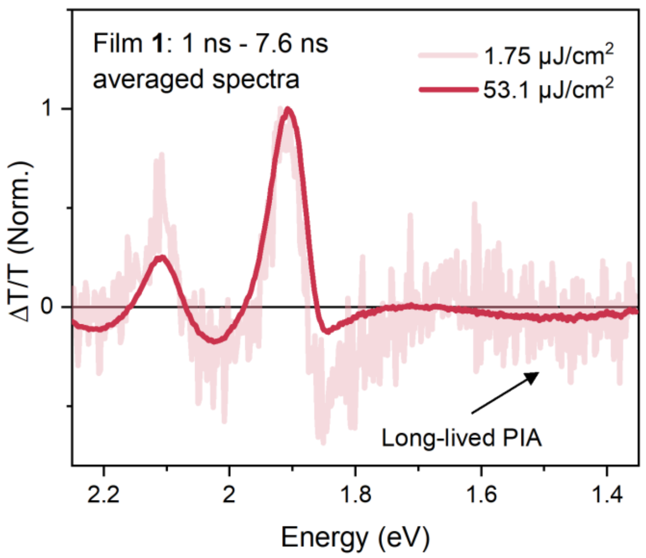

Supplementary Fig. 11 Comparison of long-lived TA spectra (averaged from 1 to 7.6 ns) between low (1.75 μJ/cm^2) and high (53.1 μJ/cm^2) pump fluence results. The black arrow indicates apparent near-infrared PIA bands both at low and high pump fluences.

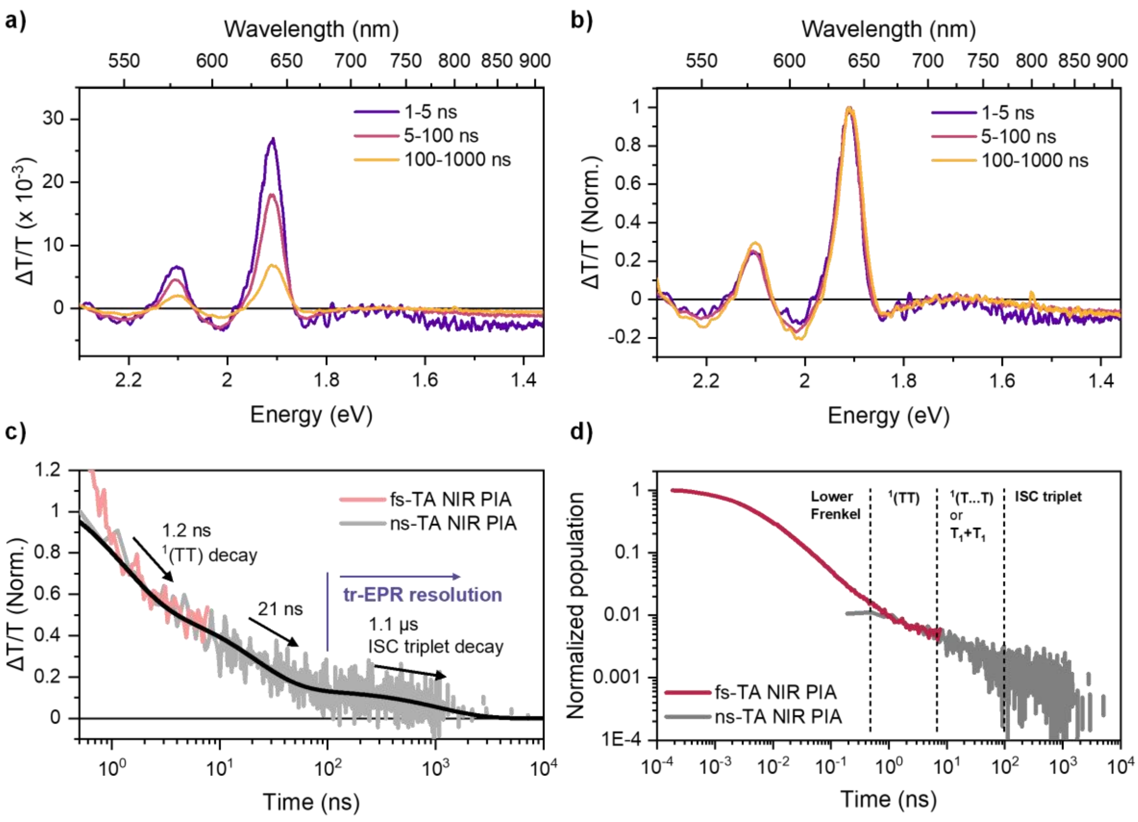

Supplementary Fig. 12 ns-TA results of film 1 after photoexcitation at 2.38 eV with 764 μJ/cm^2. **a** averaged and **b** normalized ns-TA spectra at indicated delay times. **c** Normalized ΔT/T kinetic averaged over the NIR PIA from 1.7 to 1.35 eV. The kinetic was fitted by a triexponential model (black solid line). The NIR PIA kinetic obtained from fs-TA is overlapped for comparison. **d** Normalized population kinetics estimated from combined fs- and ns-TA results. We approximately delineate several temporal regions where specific species dominate, based on the time constants from multiexponential fits.

Supplementary Note 3. ns-TA results for film 1

We performed ns-TA to get further insight into the long-lived kinetics of near-infrared (NIR) PIA in film 1 beyond the time scale accessible by fs-TA (Supplementary Fig. 12). The overall shapes of ns-TA spectra are identical to those of the spectra obtained through fs-TA (Fig. 3c): Two GSB peaks around 2.1 and 1.9 eV, overlapping PIA from 2.3 to 1.7 eV, and NIR PIA from 1.7 to 1.35 eV. Due to the possible contribution of thermal artifacts discussed in the main text, we only focused on the dynamics of the NIR PIA. The normalized ΔT/T kinetics of the NIR PIA is shown in Supplementary Fig. 12c. We were able to fit the kinetics by a triexponential model with the time constants of 1.2 ns, 21 ns, and 1.1 μs. The first time constant, 1.2 ns, is analogous to the results obtained through fs-TA and ns-PL (Fig. 3d and Supplementary Fig. 17), enabling us to assign it as the lifetime of ^{1}(TT). The last, 1.1 μs, supported by the tr-EPR results can be attributed to SO-ISC triplets. The origin of the intermediate time constant (21 ns) is ambiguous since there is no additional data available to explore the nature of this species, but we can suggest two possibilities: 1) ^{1}(T…T) or 2) free triplets, T_1+T_1. In particular, the reason why we can assign all the species observed in ns-TA as triplet-related species is based on the similarity of the spectral shapes in the entire time window. Nevertheless, regardless of the origin of the species, what we can say from the overall TA results is that ^{1}(TT) dissociation in the DPP thin film is highly inefficient if it happens at all, and the SO-ISC triplet yield is also very low judging by the substantial decay of total signal within the first few ns (Supplementary Fig. 12d). Further light could be shed on the mechanism of these longer-time processes through magnetic-field-dependent experiments to manipulate the spin evolution of TT, as suggested in the main text, but those are beyond the scope of this work.

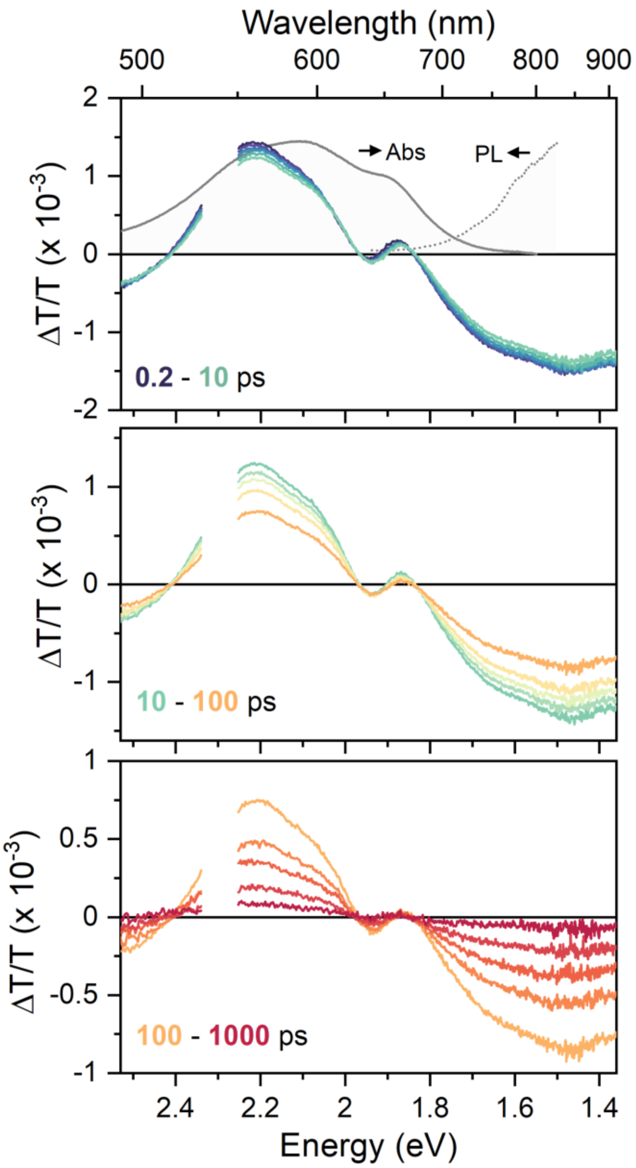

Supplementary Fig. 13 TA spectra of film 2 after photoexcitation at 2.3 eV with 1.75 μJ/cm^2. Top panel: 0.2 – 5 ps, middle panel: 5 – 200 ps, bottom panel: 200 – 1000 ps. Steady-state absorption (gray solid line) and PL (gray dotted line) are presented in the top panel for reference. TA spectra near 2.3 eV are truncated due to pump scattering.

Supplementary Note 4. Excited-state dynamics of H-like film 2

The first 12 ps kinetics just entails minor spectral changes in a part of GSB and PIA, thus suggesting relaxation processes, such as interchromophore relaxation (toward perfect sandwich geometry) or downhill energy transfer (Supplementary Fig. 13, top). There is no difference in the kinetics even when using red-edge excitation, suggesting that we can safely exclude vibrational relaxation or vibrational cooling processes (Figure 5c in the main text). In contrast, all TA spectra, regardless of GSB and PIA, simultaneously decay toward the baseline without any changes in their shape, implying ground-state recovery, during the 84 and 363 ps

kinetics (Supplementary Fig. 13, middle and bottom). Therefore, both kinetics can be ascribed to the lifetimes of different sub-ensembles in the excimer-like manifolds.

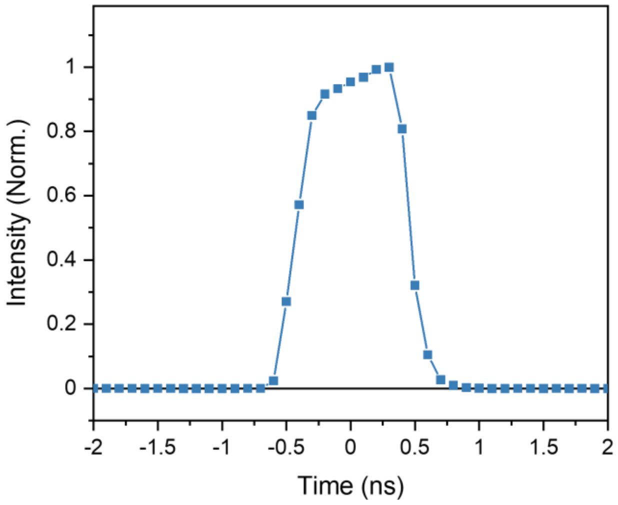

Supplementary Fig. 14 Instrument response function of TRPL experiments.

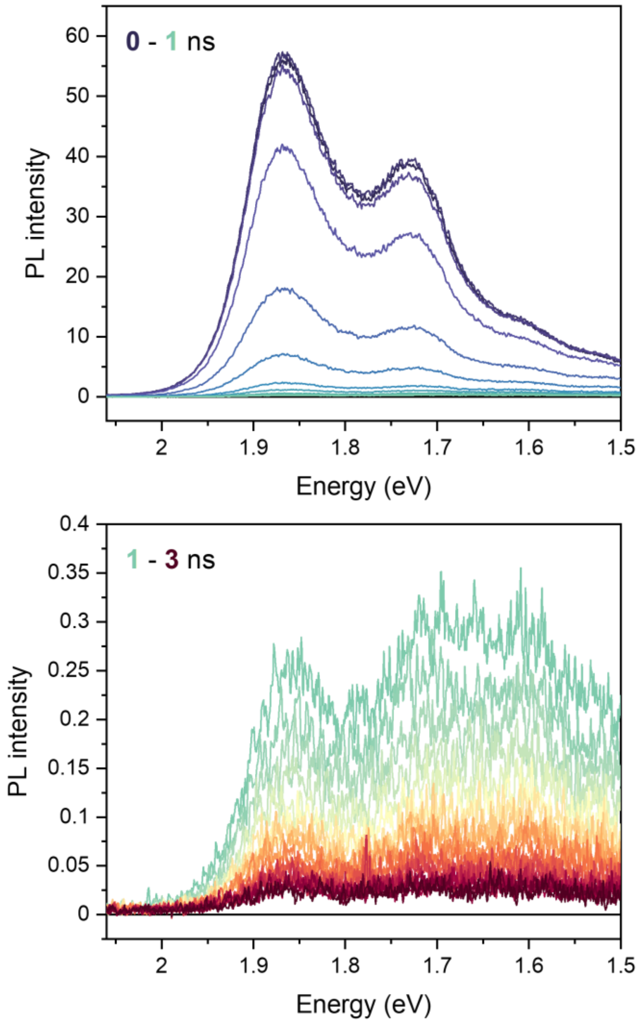

Supplementary Fig. 15 Raw TRPL spectra of film 1 after photoexcitation at 2.34 eV with 2 μJ/cm^2 in the delay range of 0 – 1 ns (top) and 1 – 3 ns (bottom).

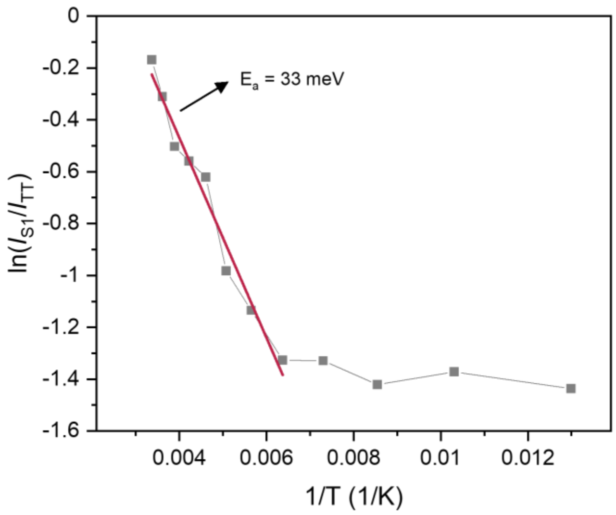

Supplementary Fig. 16 Temperature-dependent relative PL intensities between lower Frenkel and ^{1}(TT). Delayed lower Frenkel appears from 157 K, indicating the activation energy barrier of 33 meV estimated from an Arrhenius equation.

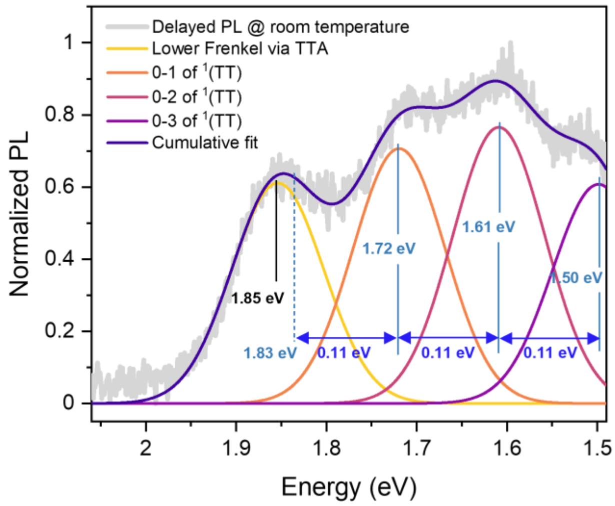

Supplementary Fig. 17 Gaussian fits for the delayed PL spectrum of film 1 at room temperature to estimate the energy level of ^{1}(TT). Here, four Gaussians are used, and it is assumed that the first peak is from lower Frenkel, and the other three peaks are from ^{1}(TT). The peak position of first Gaussian is matched with the peak position of prompt PL from the lower Frenkel. Note that three vibronic peaks are identically spaced with 0.11 eV, suggesting that the symmetry-breaking (non-totally symmetric) vibration having the energy of 0.11 eV mainly contributes to Herzberg-Teller emission. The estimated ^{1}(TT) energy level from this is 1.83 eV, which signifies that ^{1}(TT) formation from the lower Frenkel is slightly exothermic (nearly isoergic).

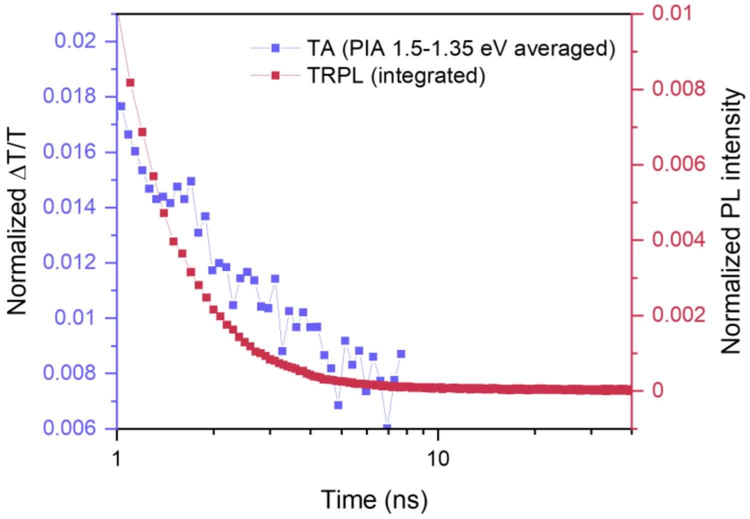

Supplementary Fig. 18 Comparison of fs-TA and TRPL kinetics of film 1 in the nanosecond delay regime. As can be seen, both kinetics are quantitatively matched with each other, indicating the decay dynamics of ^{1}(TT) with the time constant of 1.2 ns.

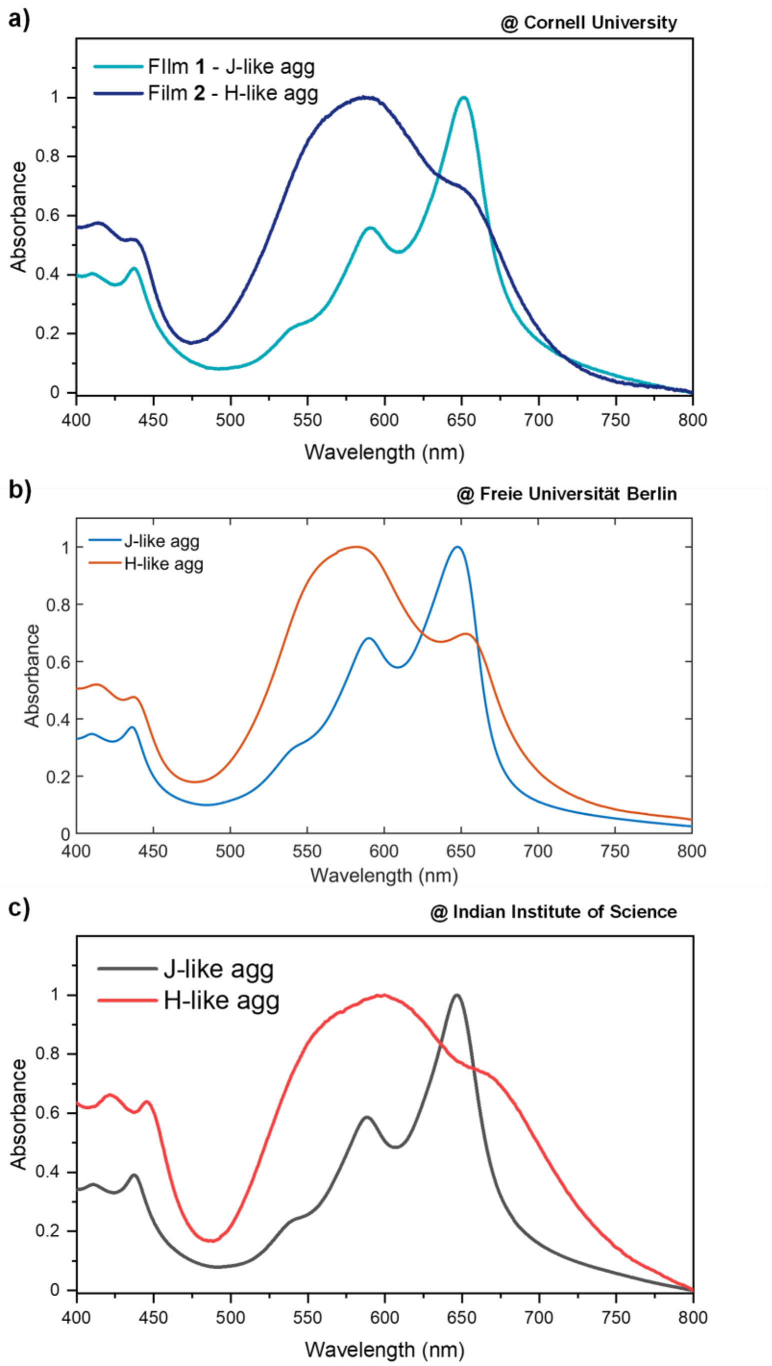

Supplementary Fig. 19 Comparison of normalized steady-state absorption spectra of J-like film 1 and H-like film 2 made in different laboratories. Time-resolved optical spectroscopic and EPR results were obtained by films shown in the top and middle panels, respectively.

Supplementary Note 5. trEPR study: orientation, temperature and excitation wavelength dependence

As part of the trEPR study on the J and H like aggregate films we also carried out an orientation study where we rotate around the long axis of the EPR tube in order to observe changes in the trEPR spectrum of the on-substrate thin films. On-substate films which have a preferential

ordering relative to the substrate can show orientation effects in trEPR. Rotating the sample results in changes in the number of molecules which have their molecular X, Y or Z axis aligned to the external magnetic field direction thereby changing the relative intensity of these canonical peaks.

The main aim of the orientation study was to attempt detection of triplet states which could correspond to singlet fission born free triplets. We therefore measured the trEPR spectrum at 50 K for both aggregate films (J and H like) with excitation at 532 nm and additionally the J like aggregate at 650 nm. The 2-dimension (2-d) map showing the trEPR spectrum as a function of time after the laser flash is shown in Supplementary Fig. 20 and the 1-dimension (1-d) field swept spectra with time-averaging to improve signal/noise in Supplementary Fig. 21. We record the trEPR spectrum at three orientations namely 0° (where the substrate plane

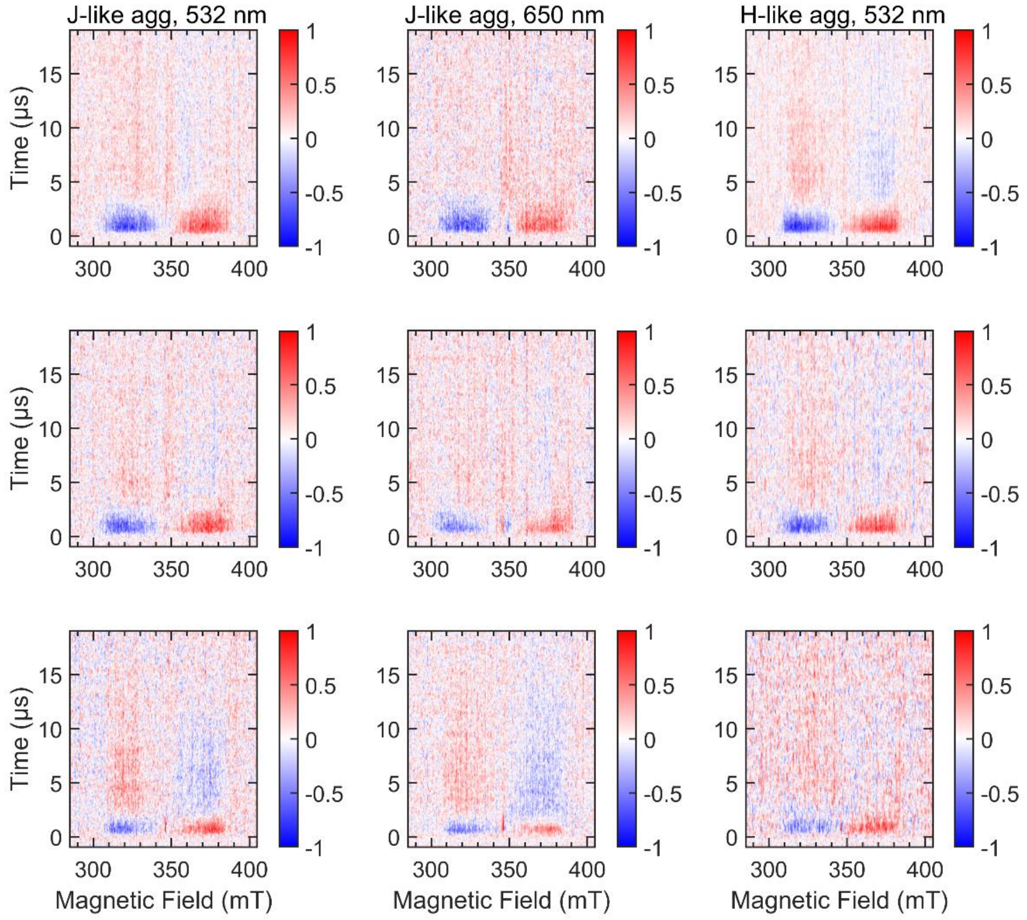

Supplementary Fig. 20 2-d trEPR plots of HR-TDPP-TEG, J and H like aggregate films measured at 50 K. Left: J like aggregate film trEPR after excitation at 532 nm (2.33 eV), Middle: after 650 nm (1.91 eV) excitation and Right: H like aggregate after excitation at 532 nm. Top to bottom: three different orientations (0°, 45°, 90°) of the substrate relative to the external magnetic field direction.

is parallel to the external magnetic field direction and perpendicular to the incident laser beam), 45° and 90° (where the substrate plane is parallel to the incident laser beam and perpendicular to the external magnetic field direction).

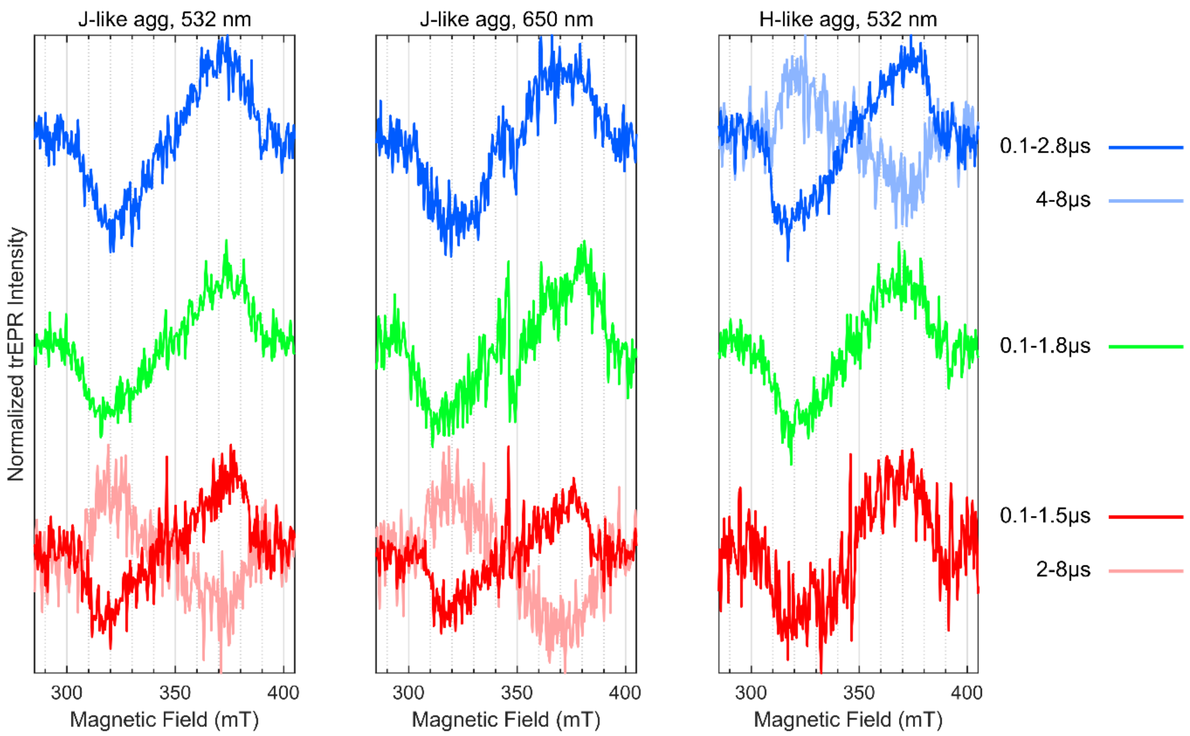

Supplementary Fig. 21 1-d time averaged slices trEPR of HR-TDPP-TEG, J and H like aggregate films measured at 50 K. Left: J like aggregate film trEPR after excitation at 532 nm, Middle: after 650 nm excitation and Right: H like aggregate after excitation at 532 nm. Top to bottom: three different orientations (0°, 45°, 90°) of the substrate relative to the external magnetic field direction. Time averaged data to increase signal/noise ratio.

We find that for both J and H like aggregate films, there is an orientation dependency where the spectral width, shape, and time evolution changes with orientation. However, even after extensive measurement averaging and for the 1-d field swept spectra also time averaging (Supplementary Fig. 21) the signal is still very weak. This makes any detailed analysis of differences/ changes in spectral shape or the nature of the partial ordering of molecules on the substrate difficult. The triplet state observed for both J and H like aggregate films, can however be described by zero-field splitting (ZFS) parameters of $D \approx 1150 - 1250$ MHz and $|E| \approx 290 - 360$ MHz. As stated in the main text the D value is smaller than reported for a TDPP triplet (TDPP without any side groups), where $D = 1550$ MHz and $|E| = 340$ MHz.[14] The smaller D parameter in our case would suggest a greater delocalization of the spin density which is in line with having TEG and/or HR side chains in HR-TDPP-TEG, not present in TDPP. Assuming a positive sign for D as was given for the TDPP recombination triplet based

on the polarization pattern observed and also further assuming an opposite sign for E as is convention, the triplet sublevel ordering from low to high energy would be T_Z, T_Y, T_X . This would mean the outer most canonical peaks in the trEPR spectrum are the T_Z followed by T_X and the inner most peak is the T_Y peaks. The E parameter was approximately determined by looking at the trEPR recorded for the J like aggregate after 650 nm excitation (0° or 45°) (Supplementary Figs. 20 and 21, middle column) where the canonical T_Y peaks can be seen due to a slightly different population of the triplet sublevels as compared to the same sample measured after excitation at 532 nm, with otherwise identical conditions. (See model simulations in Supplementary Note 6)

The orientation study of the J like aggregate at both 532 nm and 650 nm shows that at 0° orientation there is only a relatively short-lived triplet signal which decays on the few microseconds time scale. However, at 45° and more clearly at 90° orientation a longer-lived component appears which has an inverted polarization pattern compared to the early time spectrum. This suggests that with changing orientation from 0° to 90° a larger contribution of molecules, in this case with their molecular X axis (assuming $D > 0$ and $E < 0$) aligned to the external magnetic field direction, leads to observation of a change in polarization with time due to differences in the relaxation rates of each of the triplet sublevels, from eea/aae to aae/eea. The polarization pattern at the different orientations or at different times after the laser flash, all show triplet spectra which correspond to a spin orbit coupling mediated intersystem crossing (SO-ISC) mechanism and do not indicate the presence of SF born free triplets.

The reverse behaviour is observed in H-like aggregates where the longer-lived component is observed at 0° and only the shorter-lived component is observed at 90°. This suggests differences in the partial ordering/ alignment of the molecules/film relative to the substrate. However again any detailed analysis beyond this observation is not possible due to the weak signal.

The weak trEPR signal corresponding to this SO mediated ISC triplet suggests that triplet formation in both films, even by this mechanism is unfavourable. This is in line with the two proposed pathways, 1) which leads to the formation of a ^{1}(TT) pair state via SF that does not dissociate into free triplets and 2) a small number of triplets form through SO-ISC.

As mentioned in the main text we also measured both films at 20 K and 290 K mainly using 532 nm excitation. We did measure the J like aggregate film at 20 K at an orientation of 0° with also 650 nm. The 2-d trEPR plots and 1-d time averaged field sweeps are shown for both films at 20 K (Supplementary Figs. 22 and 23) and 290 K (Supplementary Figs. 24 and 25). The time and orientation behaviour for each sample remains the same between 20 K, 50 K and 290 K.

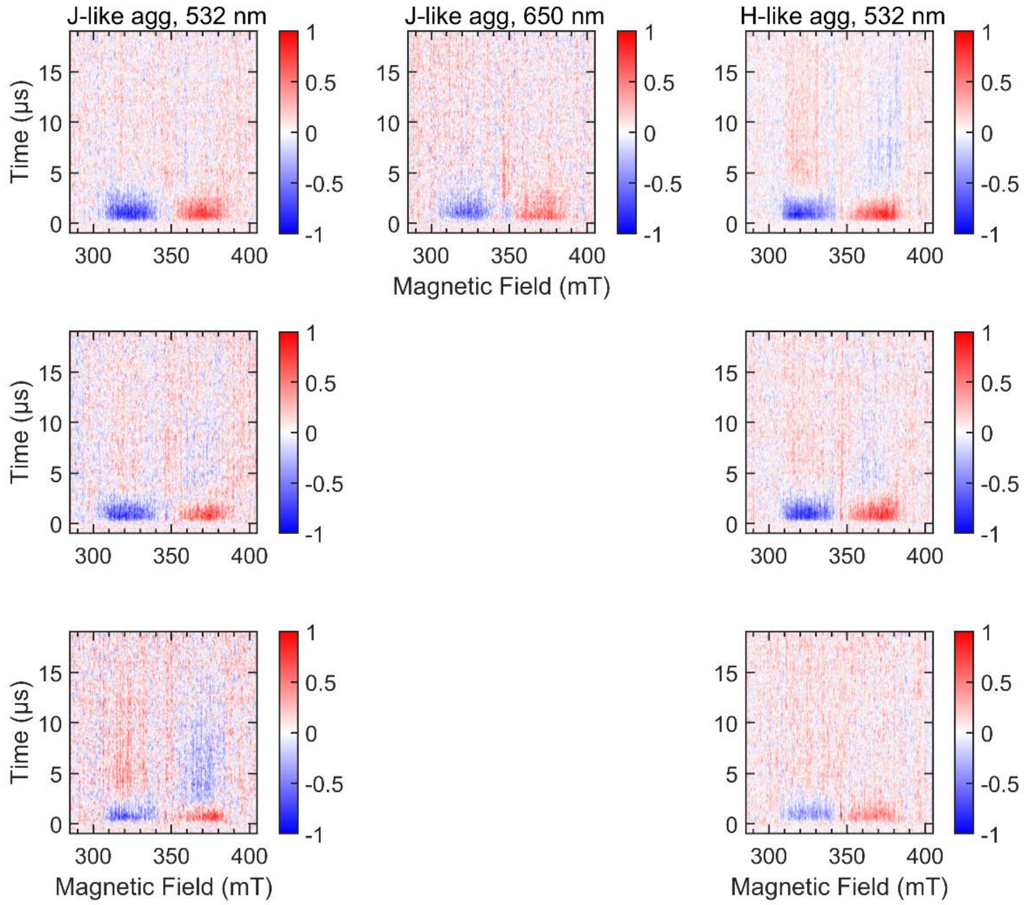

Supplementary Fig. 22 2-d trEPR plots of HR-TDPP-TEG, J and H like aggregate films measured at 20 K. Left: J like aggregate film trEPR after excitation at 532 nm, Middle: after 650 nm excitation only at 0° and Right: H like aggregate after excitation at 532 nm. Top to bottom: three different orientations (0°, 45°, 90°) of the substrate relative to the external magnetic field direction.

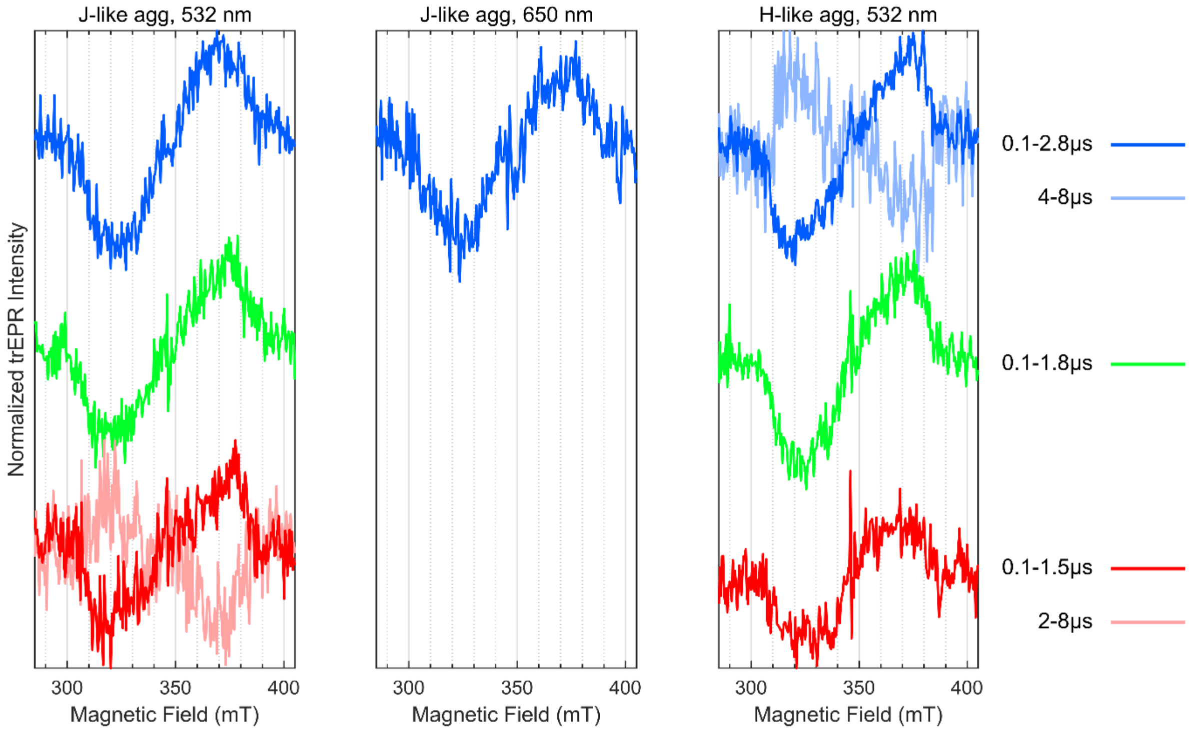

Supplementary Fig. 23 1-d time averaged slices trEPR of HR-TDPP-TEG, J and H like aggregate films measured at 20 K. Left: J like aggregate film trEPR after excitation at 532 nm, Middle: after 650 nm excitation and Right: H like aggregate after excitation at 532 nm. Top to bottom: three different orientations (0°, 45°, 90°) of the substrate relative to the external magnetic field direction. Time averaged data to increase signal/noise ratio.

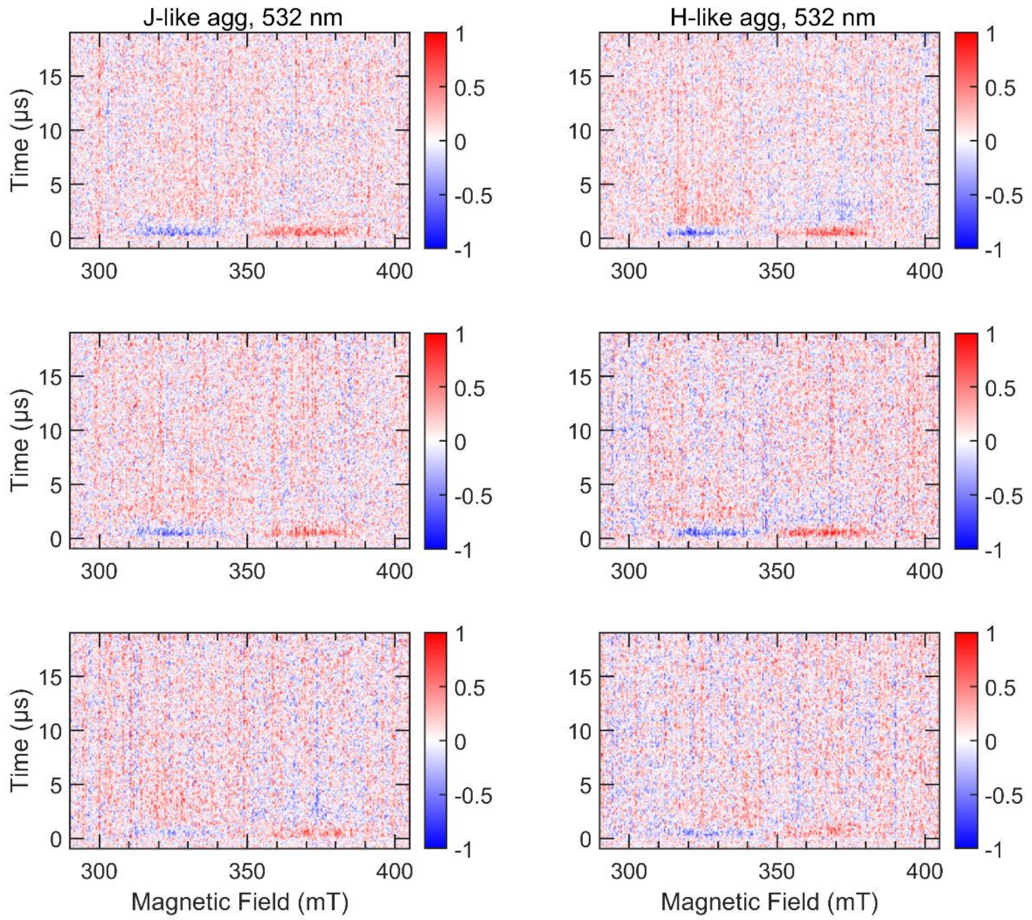

Supplementary Fig. 24 2-d trEPR plots of HR-TDPP-TEG, J and H like aggregate films measured at 290 K. Left: J like aggregate film trEPR after excitation at 532 nm and Right: H like aggregate after excitation at 532 nm. Top to bottom: three different orientations (0°, 45°, 90°) of the substrate relative to the external magnetic field direction.

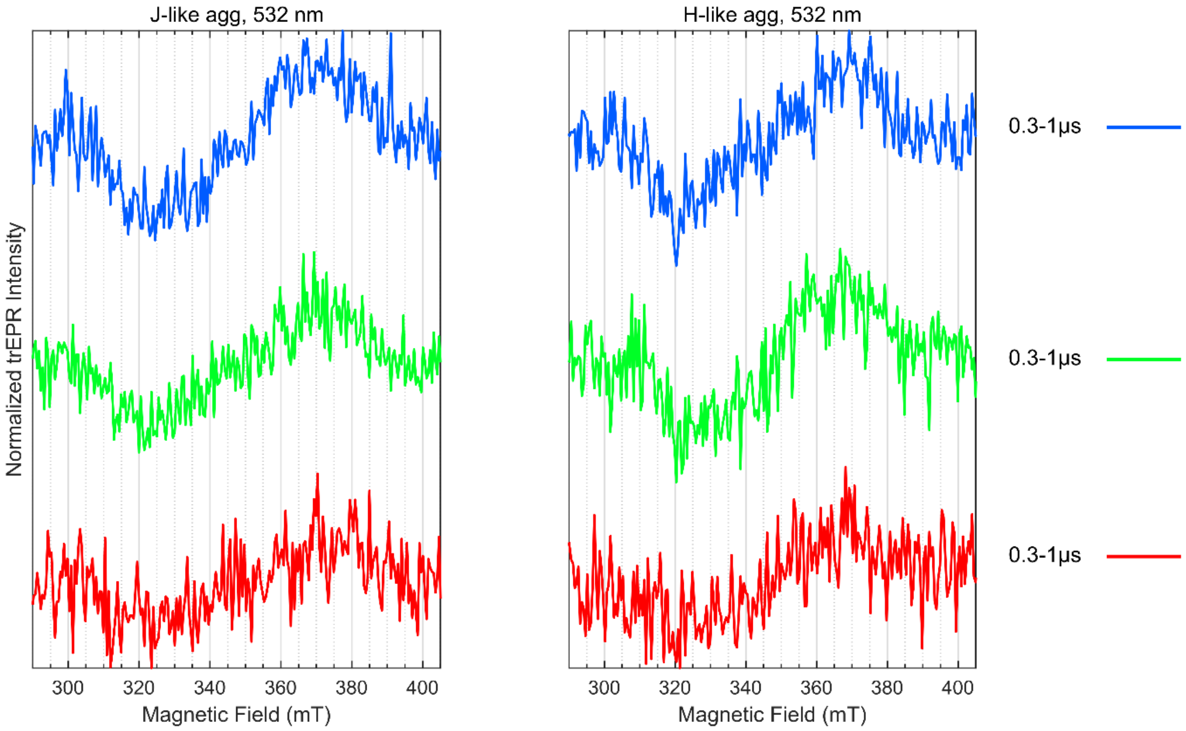

Supplementary Fig. 25 1-d time slices trEPR of HR-TDPP-TEG, J and H like aggregate films measured at 290 K. Left: J like aggregate film trEPR after excitation at 532 nm and Right: H like aggregate after excitation at 532 nm. Top to bottom: three different orientations (0°, 45°, 90°) of the substrate relative to the external magnetic field direction. Time averaged data to increase signal/noise ratio.

Supplementary Note 6. trEPR study: TrEPR triplet spectrum model simulations

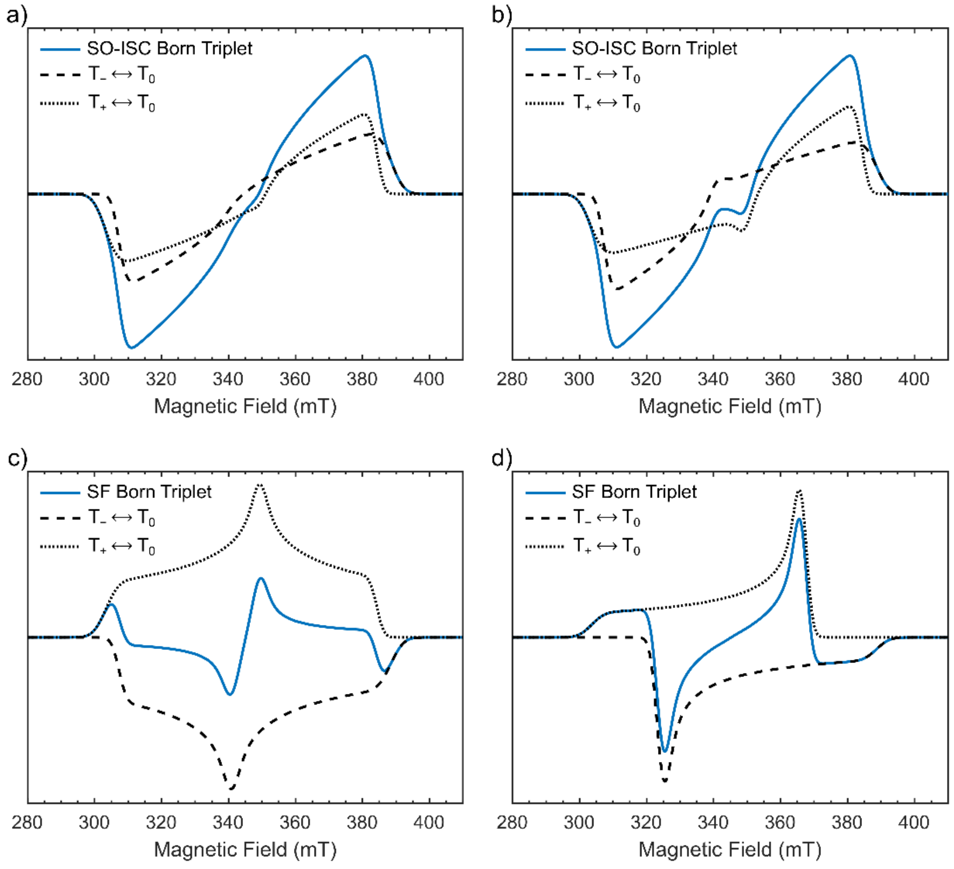

Supplementary Fig. 26 Simulated EPR spectra showing triplet state formed via SO-ISC and SF. a) and b) SO-ISC born triplet with ZFS parameters describing the triplet spectrum observed via trEPR . ***b*** shows the more pronounced inner T_Y peak due to a slightly different triplet sublvel population. c) SF born triplet with ZFS parameters of HR-TDPP-TEG. d) SF born triplet with $E = 0$ MHz, showing a more typical SF born triplet spectrum.

We use the MATLAB toolbox EasySpin[16] to simulate four model triplet EPR spectra. The first two triplet spectra (Supplementary Figs. 26a and 26b) correspond to an SO-ISC born triplet state spectrum. In both **a** and **b** we use the ZFS parameters of $D = 1200$ MHz and $E = -320$ MHz which describes the TDPP based triplet state observed for both the J and H like aggregate films. In a SO-ISC born triplet state the triplet sublevel populations are determined by their initial zero-field eigenstate populations, and here we use the relative zero-field populations of $P_X, P_Y, P_Z = 0.7, 0.3, 0$ for model spectrum **a** and $P_X, P_Y, P_Z = 0.8, 0.2, 0$ for model spectrum **b**. In both **a** and **b** the polarisation pattern is eea/aee which is clearly observed by looking at the $T_- \leftrightarrow T_0$ and $T_+ \leftrightarrow T_0$ transition, separated component spectra. The small change in relative populations of the zero-field eigenstates between simulation **a** and **b** results in a significant change in intensity of the inner most canonical (T_Y) peak. Model simulations **a**

and **b** represent the observed difference in the triplet spectrum when exciting the J like aggregate film at 532 nm and 650 nm respectively.

Next, we look at spectrum Supplementary Fig. 26c. Here the ZFS splitting parameters stay the same as for model spectra **a** and **b** however model spectrum **c** corresponds to a SF born free triplet where the high-field eigenstates are directly populated, in this case selective overpopulation of the T_0 sublevel, with a population of $P_{-1}, P_0, P_{+1} = 0, 1, 0$. The polarisation pattern is now aee/aae. The model spectrum **c** looks atypical for a SF born free triplet, this is due to the strong rhombicity of the triplet state spin distribution, i.e., large E parameter. When making the E parameter 0 MHz, the typically observed SF born triplet spectrum for e.g., TIPS-tetracene films is recovered.[17]

The model spectra simulations confirm that we observe only SO-ISC born triplet states. The parameters for each simulation are shown in Supplementary Table 3.

Supplementary Table 3. Triplet state parameters used for simulating model spectra. $g = 2.0023$ for all simulations. D strain, line broadening, in D is 200 MHz and in E 40 MHz.

Spectrum	Triplet Formation Mechanism	Polarisation Pattern	Relative Triplet Sublevel Population	D / MHz	E / MHz
a	SO-ISC	eea/eaa	$P_X, P_Y, P_Z = 0.7, 0.3, 0$	1200	-320
b	SO-ISC	eea/eaa	$P_X, P_Y, P_Z = 0.8, 0.2, 0$	1200	-320
c	SF	aee/aae	$P_{-1}, P_0, P_{+1} = 0, 1, 0$	1200	-320
d	SF	aee/aae	$P_{-1}, P_0, P_{+1} = 0, 1, 0$	1200	0

Supplementary Note 7. trEPR study: Observation of a charge transfer state at low temperatures

A charge transfer (CT) state is a cation/anion pair with exchange or dipolar spin-spin interactions. This leads to a very specific trEPR signal where an absorptive and emissive signal arises for each radical in the pair, i.e., an anti-phase doublet for each radical.[18-20] Depending on the strength of the spin-spin interactions and the g-factors of the cation and anion the absorptive and emissive signals can overlap, and the observed signal can deviate from the characteristic $e/a/e/a$ or $a/e/a/e$ spectrum.

In the case of both the J and H like aggregate films, we observe narrow signals (0.4 -1 mT wide) around $g = 2$ which correspond to a CT state (Supplementary Figs. 27 and 28). This

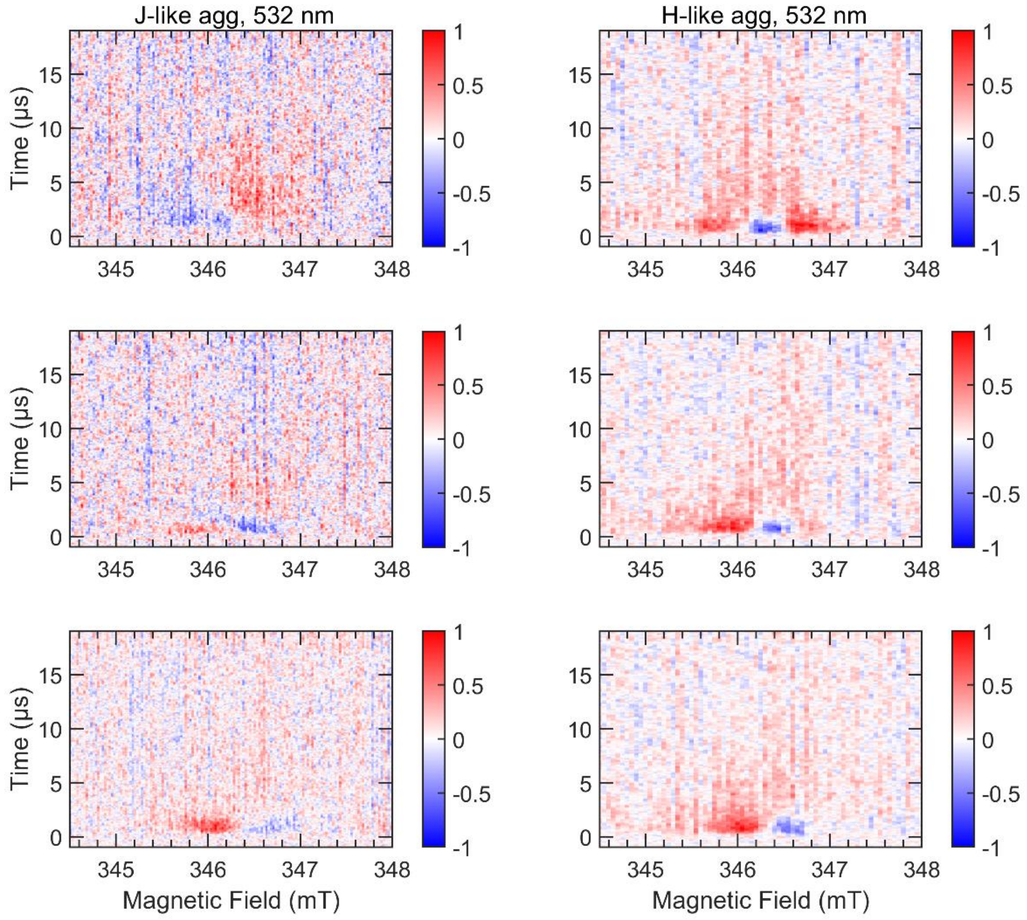

Supplementary Fig. 27 2-d trEPR plots of HR-TDPP-TEG, J and H like aggregate films measured at 20 K around g=2 showing the charge transfer state. Left: J like aggregate film trEPR after excitation at 532 nm and Right: H like aggregate after excitation at 532 nm. Top to bottom: three different orientations (0°, 45°, 90°) of the substrate relative to the external magnetic field direction.

signal is most clearly observed at 20 K. Measuring the trEPR signal around the $g = 2$ region at the three sample orientations of 0°, 45°, 90° we see that the spectrum changes.

In the case of the J-like aggregate at 0°, the narrow signal centred at $g = 2$ has a e/a polarization (it may have more features which cannot be distinguished due to low signal/noise ratio of the data) which becomes an a/e polarization at 45° and 90°. This can be explained by the change in the effective dipolar coupling which is dependent on the angle between the vector joining the dipole/exchange coupled anion cation pair and the external magnetic field direction.

In the case of the H like aggregate at 0° the narrow signal centred at $g = 2$ has a $a/e/a$ polarization, suggesting a difference in the spin-spin interaction strengths and/or difference in the cation/anion g-factors. The polarization pattern changes when moving to 45° degrees and 90° where it becomes more like an a/e polarization.

The CT state signal is only clearly visible at low temperatures and most likely is a smaller contribution than the SO-ISC triplets observed, however this is difficult to quantify.

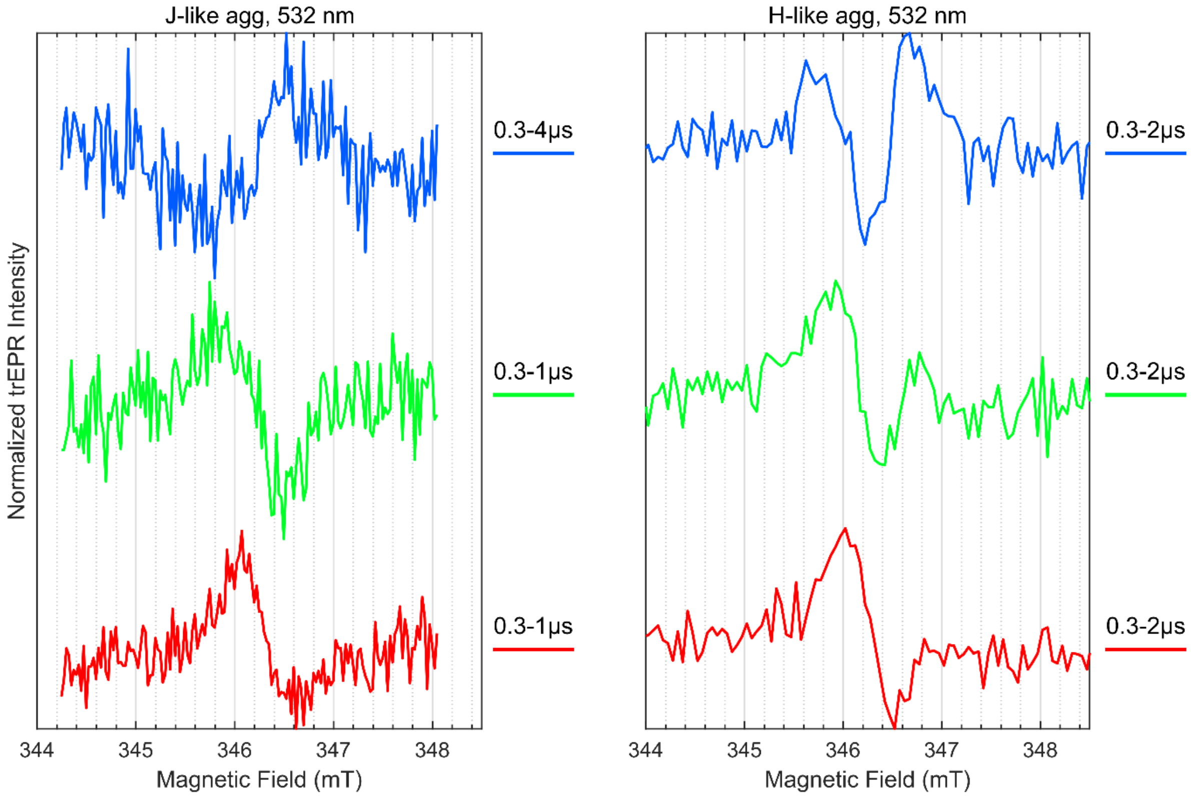

Supplementary Fig. 28 1-d time slices trEPR of HR-TDPP-TEG, J and H like aggregate films measured at 20 K around g=2 showing the charge transfer state. Left: J like aggregate film trEPR after excitation at 532 nm and Right: H like aggregate after excitation at 532 nm. Top to bottom: three different orientations (0°, 45°, 90°) of the substrate relative to the external magnetic field direction. Time averaged data to increase signal/noise ratio.

Supplementary References

1. Frisch, M. J. *et al. Gaussian 16 Rev. C.01*; Wallingford, CT, (2016).

2. Aguirre, P., Lindner, R. R. & Baker, A. J. SciNet: Lessons Learned from Building a Power-efficient Top-20 System and Data Centre You may also like The LABOCA/ACT Survey of Clusters at All Redshifts: Multiwavelength Analysis of Background Submillimeter Galaxies. *J. Phys. Conf. Ser. OPEN ACCESS* doi:10.1088/1742-6596/256/1/012026.

3. Ponce, M. *et al.* Deploying a Top-100 Supercomputer for Large Parallel Workloads: the Niagara Supercomputer. *ACM Int. Conf. Proceeding Ser.* (2019).

4. Avila Ferrer, F. J., *et al*. Insights for an Accurate Comparison of Computational Data to Experimental Absorption and Emission Spectra: Beyond the Vertical Transition Approximation. *J. Chem. Theory Comput.* **9**, 2072–2082 (2013).

5. Loken, C. *et al.* SciNet: Lessons Learned from Building a Power-efficient Top-20 System and Data Centre. *J. Phys. Conf. Ser.* **256**, 012026 (2010).

6. Baiardi, A., Bloino, J. & Barone, V. General time dependent approach to vibronic spectroscopy including franck-condon, herzberg-teller, and duschinsky effects. *J. Chem. Theory Comput.* **9**, 4097–4115 (2013).

7. Duschinsky, F. On the Interpretation of Electronic Spectra of Polyatomic Molecules. *Acta Physicochim URSS*. **7**, 551 (1937).

8. Hestand, N. J. & Spano, F. C. Expanded Theory of H- and J-Molecular Aggregates: The Effects of Vibronic Coupling and Intermolecular Charge Transfer. *Chem. Rev.* **118**, 7069–7163 (2018).

9. Knapp, E. W. Lineshapes of molecular aggregates, exchange narrowing and intersite correlation. *Chem. Phys.* **85**, 73–82 (1984).

10. Philpott, M. R. Theory of the Coupling of Electronic and Vibrational Excitations in Molecular Crystals and Helical Polymers. *J. Chem. Phys.* **55**, 2039 (2003).

11. Spano, F. C. The spectral signatures of frenkel polarons in H- And J-aggregates. *Acc. Chem. Res.* **43**, 429–439 (2010).

12. Spano, F. C. Absorption and emission in oligo-phenylene vinylene nanoaggregates: The role of disorder and structural defects. *J. Chem. Phys* **116**, 5877 (2002).

13. Ferrer, F. J. A., Cerezo, J., Stendardo, E., Improta, R. & Santoro, F. Insights for an accurate comparison of computational data to experimental absorption and emission spectra: Beyond the vertical transition approximation. *J. Chem. Theory Comput.* **9**, 2072–2082 (2013).

14. Fulton, R. L. & Gouterman, M. Vibronic Coupling. II. Spectra of Dimers. *J. Chem. Phys.* **41**, 2280 (2004).

15. Salvadori, E. *et al.* Ultra-fast spin-mixing in a diketopyrrolopyrrole monomer/fullerene blend charge transfer state. *J. Mater. Chem. A* **5**, 24335–24343 (2017).

16. Stoll, S. & Schweiger, A. EasySpin, a comprehensive software package for spectral simulation and analysis in EPR. *J. Magn. Reson.* **178**, 42–55 (2006).

17. Weiss, L. R. *et al.* Strongly exchange-coupled triplet pairs in an organic semiconductor. *Nat. Phys.* **13**, 176–181 (2017).

18. Closs, G. L., Forbes, M. D. E. & Norris, J. R. Spin-polarized electron paramagnetic

resonance spectra of radical pairs in micelles: observation of electron spin-spin interactions. *J. Phys. Chem.* **91**, 3592–3599 (1987).

19. Norris, J. R. *et al.* A general model of electron spin polarization arising from the interactions within radical pairs. *J. Chem. Phys.* **92**, 4239 (1990).

20. Hore, P. J. *et al.* Electron paramagnetic resonance of spin-correlated radical pairs in photosynthetic reactions. *Chem. Phys. Lett.* **137**, 495–500 (1987).
